# Acknowledgments

**DOI:** 10.4269/ajtmh.1104siii

**Published:** 2024-04-02

The team would like to acknowledge the support of the editorial staff at the *American Journal of Tropical Medicine and Hygiene* (AJTMH), especially Phil Rosenthal (editor-in-chief), Alison Jaeb, and Annie Calacci. Additionally, we would like to thank the guest editor, Kammerle Schneider, for overseeing the peer-review process.

Financial support: This supplement was coordinated and produced by the World Health Organization, the United States Centers for Disease Control and Prevention (CDC) and MESA, with funding from the Bill & Melinda Gates Foundation (INV-033246).

Disclosure: The views expressed herein are those of the author(s) and do not necessarily reflect the views of their institutions.

